# Supplementary material for: Role of Duplicate Genes in Robustness against Deleterious Human Mutations
Source: PLoS Genet. 2008 Mar 14;4(3):e1000014. doi: 10.1371/journal.pgen.1000014 (PMC2265532; doi:10.1371/journal.pgen.1000014)
Supplement: Figure S1 — Venn diagram showing the overlap of the three disease gene sets used in the analysis. Blue: SwissProt, green: OMIM, red: Jimenez-Sanchez G et al.. (0.03 MB DOC) [file pgen.1000014.s001.doc]

636

299

4

6

294

62

670

**Figure S1.** Venn diagram showing the overlap of the three disease gene sets used in the analysis. Blue: SwissProt, green: OMIM, red: Jimenez-Sanchez G *et al..*
