# Supplementary material for: Role of Duplicate Genes in Robustness against Deleterious Human Mutations
Source: PLoS Genet. 2008 Mar 14;4(3):e1000014. doi: 10.1371/journal.pgen.1000014 (PMC2265532; doi:10.1371/journal.pgen.1000014)
Supplement: Figure S2 — The distribution of the closest homolog sequence identities for the disease and all gene sets. (0.04 MB DOC) [file pgen.1000014.s002.doc]

**Figure S2.** The distribution of the closest homolog sequence identities for the disease and all gene sets.
