# Supplementary material for: Role of Duplicate Genes in Robustness against Deleterious Human Mutations
Source: PLoS Genet. 2008 Mar 14;4(3):e1000014. doi: 10.1371/journal.pgen.1000014 (PMC2265532; doi:10.1371/journal.pgen.1000014)
Supplement: Figure S3 — Human disease singleton genes as equally likely to have duplicate orthologs in the mouse, chicken, and zebrafish genomes as all human singleton genes. (0.02 MB DOC) [file pgen.1000014.s003.doc]

**Figure S3.** Human disease singleton genes as equally likely to have duplicate orthologs in the mouse, chicken, and zebrafish genomes as all human singleton genes.
