# Supplementary material for: Role of Duplicate Genes in Robustness against Deleterious Human Mutations
Source: PLoS Genet. 2008 Mar 14;4(3):e1000014. doi: 10.1371/journal.pgen.1000014 (PMC2265532; doi:10.1371/journal.pgen.1000014)
Supplement: Table S1 — Comparison of sequence identity of the closest homolog for the disease and all-gene sets using different BLASTP E-value cutoffs. (0.03 MB DOC) [file pgen.1000014.s004.doc]

**Table S1. Comparison of sequence identity of the closest homolog for the disease and all-gene sets using different BLASTP E-value cutoffs.**

| E-value | Mean sequence identity of the closest paralog | | p-value |
| --- | --- | --- | --- |
|  | Disease gene set | All gene set |  |
| 0.01 | 52.8% | 58.2% | 2*10^-7^ |
| 0.001 | 52.9% | 58.3% | 2*10^-7^ |
| 0.00001 | 53.1% | 58.4% | 2*10^-7^ |

Minimal alignable region: 80%.
