# Supplementary material for: Role of Duplicate Genes in Robustness against Deleterious Human Mutations
Source: PLoS Genet. 2008 Mar 14;4(3):e1000014. doi: 10.1371/journal.pgen.1000014 (PMC2265532; doi:10.1371/journal.pgen.1000014)
Supplement: Table S2 — Comparison of sequence identity of the closest homolog for the disease and all-gene sets using different cutoffs for the minimal alignable region between two sequences. (0.03 MB DOC) [file pgen.1000014.s005.doc]

**Table S2.** Comparison of sequence identity of the closest homolog for the disease and all-gene sets using different cutoffs for the minimal alignable region between two sequences.

| Alignable region | Mean sequence identity of the closest paralog | | p-value |
| --- | --- | --- | --- |
|  | Disease gene set | All gene set |  |
| 90% | 55.3% | 61.4% | 4*10^-7^ |
| 80% | 52.9% | 58.3% | 2*10^-7^ |
| 70% | 52.1% | 56.8% | 1*10^-6^ |

BLASTP E-value cutoff: 0.001.
