# Supplementary material for: Role of Duplicate Genes in Robustness against Deleterious Human Mutations
Source: PLoS Genet. 2008 Mar 14;4(3):e1000014. doi: 10.1371/journal.pgen.1000014 (PMC2265532; doi:10.1371/journal.pgen.1000014)
Supplement: Table S3 — Comparison of sequence identity of the closest homolog using different combinations of the disease and all-gene collections. (0.03 MB DOC) [file pgen.1000014.s006.doc]

**Table S3.** Comparison of sequence identity of the closest homolog using different combinations of the disease and all-gene collections.

| All-gene set | Disease gene set | Mean sequence identity of the closest paralog | | p-value |
| --- | --- | --- | --- | --- |
|  |  | All-gene set | Disease gene set |  |
| Ensembl | SwissProt | 52.9% | 58.3% | 1.6*10^-7^ |
|  | Valle paper | 52.7% | 58.3% | 1.4*10^-7^ |
|  | Morbid map | 52.9% | 58.3% | 4.9*10^-11^ |
| SwissProt | SwissProt | 52.9% | 58.0% | 4.3*10^-8^ |
|  | Valle paper | 54.7% | 58.0% | 2.7*10^-4^ |
|  | Morbid map | 54.5% | 58.0% | 1.8*10^-6^ |
