# Supplementary material for: Role of Duplicate Genes in Robustness against Deleterious Human Mutations
Source: PLoS Genet. 2008 Mar 14;4(3):e1000014. doi: 10.1371/journal.pgen.1000014 (PMC2265532; doi:10.1371/journal.pgen.1000014)
Supplement: Table S4 — Comparison of sequence identity of the closest homolog for the disease and all genes in different GO slim categories. (0.12 MB DOC) [file pgen.1000014.s007.doc]

**Table S4.** Comparison of sequence identity of the closest homolog for the disease and all genes in different GO slim categories

| **Molecular function** | | | | | | | | |
| --- | --- | --- | --- | --- | --- | --- | --- | --- |
| **GO ID** | **GO term** | **Number of genes** | | **Mean sequence identity (%)** | | **1-sided Wilcoxon’s test** | **Critical value (fdr=5%)** | **Rank** |
|  |  | **disease** | **all** | **disease** | **all** |  |  |  |
| **GO:0016491** | **oxidoreductase activity** | **50** | **354** | **47.7** | **55.4** | **0.0018** | **0.0075** | **8** |
| **GO:0005488** | **Binding** | **361** | **4700** | **53.1** | **56.2** | **0.0031** | **0.0094** | **10** |
| **GO:0009055** | **electron carrier activity** | **6** | **69** | **35.0** | **56.4** | **0.0032** | **0.0104** | **11** |
| **GO:0005198** | **structural molecule activity** | **34** | **334** | **59.9** | **69.5** | **0.0080** | **0.0132** | **14** |
| **GO:0003824** | **Catalytic activity** | **227** | **2395** | **53.6** | **56.8** | **0.0107** | **0.0142** | **15** |
| GO:0030234 | enzyme regulator activity | 20 | 289 | 46.4 | 54.5 | 0.0161 | 0.0160 | 17 |
| GO:0004871 | signal transducer activity | 86 | 1331 | 52.0 | 56.0 | 0.0191 | 0.0170 | 18 |
| GO:0004872 | receptor activity | 72 | 1105 | 51.3 | 55.3 | 0.0258 | 0.0198 | 21 |
| GO:0005515 | protein binding | 175 | 2144 | 52.9 | 55.6 | 0.0692 | 0.0226 | 24 |
| GO:0016874 | ligase activity | 9 | 121 | 48.9 | 58.7 | 0.0749 | 0.0236 | 25 |
| GO:0045182 | translation regulator activity | 1 | 35 | 33.7 | 69.3 | 0.0889 | 0.0245 | 26 |
| GO:0016209 | antioxidant activity | 5 | 25 | 46.6 | 56.7 | 0.1003 | 0.0264 | 28 |
| GO:0016740 | transferase activity | 76 | 837 | 55.3 | 57.4 | 0.1688 | 0.0311 | 33 |
| GO:0003676 | nucleic acid binding | 51 | 1197 | 52.9 | 56.6 | 0.2145 | 0.0340 | 36 |
| GO:0015075 | ion transmembrane transporter activity | 59 | 397 | 56.6 | 57.1 | 0.2706 | 0.0368 | 39 |
| GO:0016301 | kinase activity | 42 | 407 | 59.5 | 60.3 | 0.2813 | 0.0377 | 40 |
| GO:0005215 | transporter activity | 84 | 680 | 54.7 | 55.5 | 0.2901 | 0.0387 | 41 |
| GO:0015267 | channel activity | 29 | 217 | 57.5 | 58.4 | 0.3283 | 0.0396 | 42 |
| GO:0016787 | hydrolase activity | 88 | 1030 | 55.1 | 56.7 | 0.3509 | 0.0406 | 43 |
| GO:0030528 | transcription regulator activity | 41 | 514 | 52.3 | 52.9 | 0.4141 | 0.0415 | 44 |
| GO:0016829 | lyase activity | 10 | 70 | 52.7 | 53.2 | 0.4393 | 0.0443 | 47 |
| GO:0016853 | isomerase activity | 6 | 68 | 62.6 | 60.5 | 0.5746 | 0.0462 | 49 |
| GO:0003774 | motor activity | 9 | 77 | 67.3 | 61.6 | 0.7372 | 0.0481 | 51 |
| GO:0004386 | helicase activity | 1 | 33 | 79.6 | 59.1 | 0.7622 | 0.0491 | 52 |
|  |  |  |  |  |  |  |  |  |
| **Biological process** | | | | | | | | |
| **GO ID** | **GO term** | **Number of genes** | | **Mean sequence identity (%)** | | **1-sided Wilcoxon’s test** | **Critical value (fdr=5%)** | **Rank** |
|  |  | **disease** | **all** | **disease** | **All** |  |  |  |
| **GO:0050896** | **response to stimulus** | **96** | **1165** | **48.5** | **57.3** | **<0.0001** | **0.0019** | **2** |
| **GO:0008152** | **metabolic process** | **283** | **3392** | **52.5** | **57.4** | **0.0001** | **0.0047** | **5** |
| **GO:0009987** | **cellular process** | **353** | **5223** | **53.0** | **57.1** | **0.0001** | **0.0057** | **6** |
| **GO:0006118** | **electron transport** | **25** | **171** | **45.3** | **55.2** | **0.0035** | **0.0113** | **12** |
| **GO:0009058** | **biosynthetic process** | **49** | **518** | **54.2** | **62.2** | **0.0075** | **0.0123** | **13** |
| GO:0043170 | macromolecule metabolic process | 186 | 2604 | 54.1 | 57.9 | 0.0205 | 0.0179 | 19 |
| GO:0007154 | cell communication | 136 | 2062 | 53.8 | 57.0 | 0.0208 | 0.0189 | 20 |
| GO:0006810 | transport | 123 | 1172 | 53.2 | 56.8 | 0.0267 | 0.0208 | 22 |
| GO:0006519 | amino acid and derivative metabolic process | 29 | 122 | 50.0 | 56.0 | 0.0513 | 0.0217 | 23 |
| GO:0046903 | secretion | 8 | 136 | 48.8 | 58.0 | 0.0947 | 0.0255 | 27 |
| GO:0008219 | cell death | 33 | 282 | 47.3 | 51.3 | 0.1050 | 0.0274 | 29 |
| GO:0009056 | catabolic process | 39 | 294 | 53.6 | 56.8 | 0.1125 | 0.0283 | 30 |
| GO:0007610 | behavior | 11 | 114 | 45.0 | 52.1 | 0.1259 | 0.0292 | 31 |
| GO:0030154 | cell differentiation | 55 | 517 | 50.1 | 52.1 | 0.1992 | 0.0330 | 35 |
| GO:0007275 | multicellular organismal development | 116 | 722 | 53.2 | 54.9 | 0.2270 | 0.0349 | 37 |
| GO:0050789 | regulation of biological process | 123 | 1638 | 52.7 | 54.4 | 0.2465 | 0.0358 | 38 |
| GO:0006139 | nucleobase, nucleoside, nucleotide and nucleic acid metabolic process | 73 | 1308 | 55.5 | 57.0 | 0.4187 | 0.0434 | 46 |
| GO:0006928 | cell motility | 15 | 141 | 61.9 | 56.5 | 0.8334 | 0.0500 | 53 |
|  |  |  |  |  |  |  |  |  |
| **Cellular component** | | | | | | | | |
| **GO ID** | **GO term** | **Number of genes** | | **Mean sequence identity (%)** | | **1-sided Wilcoxon’s test** | **Critical value (fdr=5%)** | **Rank** |
|  |  | **disease** | **all** | **Disease** | **All** |  |  |  |
| **GO:0005737** | **cytoplasm** | **180** | **1592** | **50.8** | **58.1** | **<0.0001** | **0.0009** | **1** |
| **GO:0005623** | **cell** | **398** | **5522** | **52.1** | **56.5** | **<0.0001** | **0.0028** | **3** |
| **GO:0005622** | **intracellular** | **258** | **3370** | **52.1** | **57.7** | **<0.0001** | **0.0038** | **4** |
| **GO:0005576** | **extracellular region** | **50** | **504** | **44.7** | **53.1** | **0.0010** | **0.0066** | **7** |
| **GO:0005615** | **extracellular space** | **16** | **205** | **42.0** | **55.8** | **0.0025** | **0.0085** | **9** |
| GO:0016020 | membrane | 237 | 2950 | 52.3 | 55.0 | 0.0156 | 0.0151 | 16 |
| GO:0005634 | nucleus | 71 | 1467 | 52.9 | 56.4 | 0.1544 | 0.0302 | 32 |
| GO:0005694 | chromosome | 5 | 122 | 54.9 | 66.6 | 0.1811 | 0.0321 | 34 |
| GO:0005578 | proteinaceous extracellular matrix | 12 | 115 | 49.8 | 49.9 | 0.4168 | 0.0425 | 45 |
| GO:0009986 | cell surface | 6 | 47 | 57.3 | 57.6 | 0.4944 | 0.0453 | 48 |
| GO:0005941 | unlocalized protein complex | 3 | 10 | 61.3 | 60.2 | 0.6001 | 0.0472 | 50 |

*GO slim terms not included in the analysis

3 root categories: 0003674, 0008150, 0005575.

1 category which include only known disease genes: 0008402.

Categories which do not include any known disease genes: 0006944, 0009405, 0043062, 0008565, 0008907, 0030188, 0030312.

**. The information related to significant categories is given using bold fonts.
