# Supplementary material for: Role of Duplicate Genes in Robustness against Deleterious Human Mutations
Source: PLoS Genet. 2008 Mar 14;4(3):e1000014. doi: 10.1371/journal.pgen.1000014 (PMC2265532; doi:10.1371/journal.pgen.1000014)
Supplement: Table S5 — Comparison of the Similarity of Tissue Expression (STE) between the disease and all gene sets for sequences with various sequence identities of the closest homolog. (0.03 MB DOC) [file pgen.1000014.s008.doc]

**Table S5. Comparison of the Similarity of Tissue Expression (STE) between the disease and all gene sets for sequences with various sequence identities of the closest homolog.**

| Sequence identity to the closest homolog | Average for disease genes | Average for all genes | Wilcoxon’s two sample P-value |
| --- | --- | --- | --- |
| 0.3 - 0.4 | 0.169 | 0.180 | 3*10^-2^ |
| 0.4 – 0.5 | 0.187 | 0.188 | 3*10^-2^ |
| 0.5 – 0.6 | 0.172 | 0.188 | 1*10^-3^ |
| 0.6 – 0.7 | 0.169 | 0.214 | 3*10^-4^ |
| 0.7 – 0.8 | 0.178 | 0.242 | 2*10^-2^ |
| 0.8 – 0.9 | 0.278 | 0.351 | 0.5 |
| > 0.9 | 0.279 | 0.415 | 0.2 |
| All gene pairs | 0.18 | 0.21 | 6*10^-9^ |
