# Supplementary material for: Role of Duplicate Genes in Robustness against Deleterious Human Mutations
Source: PLoS Genet. 2008 Mar 14;4(3):e1000014. doi: 10.1371/journal.pgen.1000014 (PMC2265532; doi:10.1371/journal.pgen.1000014)
Supplement: Text S1 — Investigating the duplicability of human genes. (0.03 MB DOC) [file pgen.1000014.s009.doc]

**Investigating the duplicability of human genes.**

Our approach for investigating the duplicability of human genes follows the analysis by He *et al.* [30]. For all human singleton genes, we obtained their orthologs in the mouse, chicken and zebrafish genomes from the Ensembl database. Importantly, the orthologs of human singleton genes may or may not have duplicates in their corresponding genomes. Genes with only one-to-null or one-to-one orthologs in the three other genomes are termed singleton genes with singleton orthologs. For genes with one-to-many orthologs, we distinguished two types of situations: 1.) non-human species-specific duplication and 2.) human-specific gene loss. For example, in mouse-specific duplication, the divergence time between the mouse paralogs is less than that between the human gene and its mouse orthologs. On the other hand, in a case of human-specific gene loss, the divergence time between the mouse paralogs is greater than that between the human gene and its mouse orthologs. We identified genes with non-human species duplication in any of the three considered genomes; these are termed singleton genes with duplicated orthologs.

Numbers of orthologs of human singleton genes identified in each of the three considered genomes:

Disease singleton genes with singleton orthologs in mouse / chicken / zebrafish: 336 / 260 / 212. Disease singleton genes with duplicated orthologs in mouse / chicken / zebrafish: 5 / 1 / 26

Human singleton genes with singleton orthologs in mouse / chicken / zebrafish: 5630 / 4206 / 3057. Human singleton genes with duplicated orthologs in mouse / chicken / zebrafish: 99 / 4 / 366
